# Vitamin D_3_ Supplementation in Drinking Water Prior to Slaughter Improves Oxidative Status, Physiological Stress, and Quality of Pork

**DOI:** 10.3390/antiox9060559

**Published:** 2020-06-26

**Authors:** Ana I. Rey, José Francisco Segura, David Castejón, Encarnación Fernández-Valle, Mª Isabel Cambero, Luis Calvo

**Affiliations:** 1 Department of Animal Production, Facultad de Veterinaria, Universidad Complutense de Madrid, Avda. Puerta de Hierro s/n., 28040 Madrid, Spain; josesegu@ucm.es; 2 Unidad de RMN (CAI de Bioimagen), Universidad Complutense de Madrid. Pº de Juan XXIII, 1, 28040 Madrid, Spain; dcastejon@ucm.es (D.C.); evalle@ucm.es (E.F.-V.); 3 Department Section of Food Technology, Facultad de Veterinaria, Universidad Complutense de Madrid, Avda. Puerta de Hierro s/n., 28040 Madrid, Spain; icambero@ucm.es; 4 Incarlopsa. I+D department. Ctra. N-400 km. 95400, 16400 Cuenca, Spain; luiscalvo@incarlopsa.es

**Keywords:** vitamin D_3_, drinking water, lipid oxidation, free-fatty acids, drip loss, proteolysis, pig

## Abstract

The aims of this study were to investigate the effect of vitamin D_3_ administration in drinking water during lairage time prior to slaughter on physiological stress, oxidative status, and pork quality characteristics. Two experiments were carried out. The first one was performed to examine the effect of vitamin D_3_ supplementation in drinking water, and the second one to check the effect of supplementation dose (500,000 IU/L vs. 700,000 IU/L). Serum calcium concentration was greater in pigs receiving vitamin D_3_ in water when compared to the control group. In experiment 1, a 40% α-tocopherol increase in meat from the group supplemented with vitamin D_3_ (500,000 IU/animal) was observed, that resulted in a tendency of decreased meat malondialdehyde (MDA) values at days 5 and 8 after refrigerated storage. In experiment 2, since water intake was higher (800,000 IU and 1,120,000 IU/animal of vitamin D_3_ consumption) effects on oxidative status were more profound and vitamin D_3_ supplementation increased serum α-tocopherol and decreased cortisol and serum TBARS. These effects were also observed in meat; TBARS levels were decreased after 3 days of refrigerated storage. In both experiments meat from pigs that received vitamin D_3_ in drinking water had a lower proportion of total free-polyunsaturated fatty acids (mainly n-6) when compared to the unsupplemented pigs, and these were positively correlated with TBARS production at day 5 of refrigerated storage (*r* = 0.53 and 0.38 for experiments 1 and 2, respectively). Meat from pigs receiving vitamin D_3_ in water showed reduced cohesiveness, gumminess, and chewiness values compared to the control group. The magnetic resonance imaging study of muscle confirmed the effects on water retention with lesser transverse relaxation time in pigs supplemented with vitamin D_3_. No vitamin D_3_ dose effect was observed, apart from muscle α-tocopherol concentration that was higher in pigs supplemented with 700,000 compared to those supplemented with 500,000 UI/L.

## 1. Introduction

Vitamin D_3_ is a liposoluble compound required for the absorption of calcium and phosphorus in the intestines. It is biologically inert and must be metabolized to 25-hydroxyvitamin D_3_ in the liver and then to 1α, 25-dihydroxyvitamin D_3_ in the kidney in order to be activated [1]. Recent studies confirm the important physiological functions of this vitamin, which has been related to immunity control [2] and its deficiency could lead to several health disorders [3]. Moreover, it has been reported that this vitamin is involved in stress regulation, due to the similarities of its nuclear receptor ligand to those of corticosteroids, which act on the hypothalamus–pituitary–adrenal axis [4]. The similarities between these receptors mean that both substances compete and one or the other can be used in case of intoxication or overdose [5]. Consequently, this vitamin could be a potential mechanism used in intensive production practices that cause stress and affect the quality of the products. Recently, the protective effect of vitamin D against oxidative stress [6] has been reported in humans, as well as its direct association with increased glutathione-peroxidase levels, which is one of the main antioxidant enzymes in the organism [7]. However, such effects and the persistence of possible antioxidant properties after slaughter have been scarcely studied in animals and results are not conclusive.

Additionally, one of the dietary strategies based on vitamin D supplementation in animal diets is its application to improve tenderness as a result of enhanced post-mortem proteolysis [8]. This is based on the activation of the Ca^2+^ ions by vitamin D_3_, which are responsible for activation of enzymes from the calpain group that mediates the degradation of myofibrillar and cytoskeletal proteins post-mortem [9]. In addition, vitamin D may indirectly modify other meat quality characteristics such as drip loss since the enhancement in muscle proteolysis has been associated with increased water retention [10] and juiciness.

In the majority of the studies on vitamin D_3_ supplementation, high doses are added into the diets, and there is a lack of information of the possible effects when administered in drinking water at high concentrations for a short period. In a previous study, one-time dose of 40,000 IU resulted in fewer piglets losing weight during the first 7 days post-weaning [11]. Considering that supra-nutritional doses may retard growth [12], and that stress prior to slaughter in fasting conditions may modify the animal’s oxidative status and, consequently, the stability and nutrient value of the products, its administration under these special conditions deserves more attention. Accordingly, drinking water could be an interesting vehicle for providing short-term vitamin D_3_ supplementation to fasted pigs without compromising growth and with possible beneficial effects on meat quality. 

Therefore, the objectives of this study were to investigate the effect of vitamin D_3_ administration—added to drinking water at different doses during lairage prior to slaughter—on physiological stress, oxidative status, and meat quality characteristics such as pH, drip loss, muscle composition, meat stability, and tenderness.

## 2. Materials and Methods

All experimental procedures performed in this study complied with Spanish policy for Animal Protection (RD53/2013), which is in accordance with the European Union Directive 2010/63/UE on the protection of animals used for research and with the research committee of the Veterinary Faculty of Complutense University of Madrid.

### 2.1. Animals, Slaughter, and Sample Collection

Experiment 1. One hundred female pigs (Large White x Landrace) housed in an environmentally controlled farm (Copiso, Centenera del Campo, Soria, Spain) received the same basal diet during fattening which was formulated according to NRC requirements for pigs (Table S1). During fattening, feed and water were ad libitum provided. At the end of fattening phase, pigs were sent to a commercial slaughterhouse (Incarlopsa, Tarancón, Cuenca, Spain) and located in four different boxes (25 animals per box) during lairage time (4 h, autumn with a maximum temperature of 19 °C). Water was provided ad libitum in two different tanks that were fitted with a water meter, agitator, and a reactor system: one tank contained the Vitamin D_3_ supplement at 500,000 IU/L (VIT-500), and the other was not supplemented (Control). 

Experiment 2. Barrows Danbreed pigs (*n* = 150) were maintained in the same farm conditions as described in experiment 1, and received the diet presented in Table S1. In this experiment, animals were located in six different boxes in the slaughterhouse (Incarlopsa, Tarancón, Cuenca, Spain) with a density of 25 animals per box. During lairage time (4 h, autumn with a maximum temperature of 27 °C), the pigs received water ad libitum from three different tanks that were fitted with a water meter, agitator, and a reactor system: one tank contained a lower dose of Vitamin D_3_ supplement (500,000 IU/L) (VIT-500), and the second contained a higher dose of Vitamin D_3_ supplement (700,000 IU/L) (VIT-700). The third one was not supplemented (Control). 

In both experiments, each deposit (1000 L of total capacity, filling up to 720 L) provided water to two of the different boxes that contained the experimental animals, and Vitamin D_3_ (APSAVIT-D_3_ 500) was provided in solid form by Pintaluba S.A. (Reus, Spain). Thus, in experiment 1, water was distributed to a total of four experimental boxes (two lines from the tank with the supplementation and two lines from the tank without the supplementation); and in experiment 2, water was distributed to a total of six experimental boxes (two lines from the tank with 500,000 IU/L; two lines from the tank with 700,000 IU/L, and two lines from the tank without supplementation). For the preparation of the experimental mixture, the pure compound APSAVIT-D_3_ (500,000 UI D_3_/g powder) was directly added in small amounts to the tank with stir mode on. The process was repeated several times until the desired dose was reached (a total of 720 g per 500,000 IU D_3_/L or 1000 g per 700,000 IU D_3_/L of APSAVIT-D_3_ were used). During the experimental time, the agitator operated continuously to maintain the vitamin D in suspension. The amount of water consumed was measured by subtracting the water meter readings at the start and end of the trial (lairage time). The water intake per pig was averaged taking into account total consumption and the number of pigs per box. The average intake of water per animal was approximately 1.1 L in experiment 1 and 1.6 L in experiment 2, representing a consumption of approximately 500,000 IU/animal in experiment 1 and 800,000 IU/animal (low-supplemented pigs) or 1,120,000 IU/animal (high-supplemented pigs) in experiment 2. Sucking drinkers (two per box) were located in parallel at 0.6 m above the ground on the outer line of the box. The total fasting time (farm, transport and lairage) before slaughter was approximately 17 h for both experimental assays. Animals were stunned, slaughtered by CO_2_, slaughtered and left bleeding, and carcasses were chilled at 4 °C (cooling air flow 1m/s; 90% relative humidity) for 24 h. At the slaughter time, blood samples from the jugular vein were collected in vacuum tubes for a total of 20 animals (*n* = 10 per treatment) in experiment 1, and 24 (*n* = 8 per treatment) in experiment 2. All blood samples were immediately placed on ice after collection. The serum was then separated by centrifugation at 600× *g* for 10 min at 4 °C and the supernatant was kept in a freezer at −80 °C until analyses. The ultimate pH value was recorded at 40 h on muscle (pHxK21; NWK Binar, Puergen, DE). *Longissimus dorsi* muscle was removed and samples of approximately 15 cm in size were collected at the level of the last rib (*n* = 10 per treatment in experiment 1; and *n* = 8 per treatment in experiment 2). Fresh muscle from one chop was used for drip loss and TBARS measurements, and an additional chop was stored in individual, vacuum-packed plastic bags at −20 °C until further analyses that were carried out within the next month. In addition, malondialdehyde (MDA) and α-tocopherol were measured for the evaluation of oxidative status of serum. For the nuclear magnetic resonance study (experiment 2), an additional chop from fresh muscle was taken (*n* = 6 per treatment) and samples were immediately transported under refrigeration to the Nuclear Magnetic Resonance Unit (CAI of Bioimagen; Complutense University, Madrid, Spain).

### 2.2. Laboratory Analyses

#### 2.2.1. Serum Samples

Cortisol was determined using chemiluminescent immunoassay technology following manufacturer instructions (Shenzhen Mindray Bio-Medical Electronics Co., Ltd., Shenzhen, China). In addition, MDA and α-tocopherol levels were measured for the evaluation of oxidative status of serum. MDA was determined spectrophotometrically after the addition of perchloric acid to precipitate sample proteins [13]. Results were expressed as μmoles MDA (malondialdehyde)/L serum. The α-tocopherol concentration in serum from experimental pigs was quantified by direct extraction [14]. Results were expressed as µg of α-tocopherol per mL of serum. Vitamin D (25-OH) was quantified by chemiluminescent immunoassay technology using a Liaison analyzer (DiaSorin Gerenzano, Italy) and calcium was measured with a spectrophotometrical kit following manufacturer instructions (Shenzhen Mindray Bio-Medical Electronics Co., Ltd., Shenzhen, China). Serum analyses were performed in duplicate.

#### 2.2.2. Drip Loss in Muscle Samples

Drip loss was measured by the bag method [15]. Fresh meat samples (1 cm^3^ size) were put inside of a mesh that was covered by a plastic bag and placed under refrigerated conditions (4 °C). Samples were weighed at the beginning and again after 72 h of storage. Drip loss was calculated by difference between initial and final weights and expressed as a percentage of the initial weight.

#### 2.2.3. TBARS Analysis in Muscle Samples

The malondialdehyde (MDA) measurement was determined by the thiobarbituric acid reactive substances (TBARS) test. Chops (40 g) were placed on polystyrene trays that were over-wrapped with an oxygen permeable (6000–8000 mL O_2_/m^2^/24 h at STP) PVC wrap and kept at 4 °C under fluorescent light (616 lux). Measurements were performed on days 0, 3, 5, and 8 [16]. Meat samples were homogenized in a tube including a solution of perchloric acid (3.83% v/v) and BHT (4.2% in ethanol) in a mixer mill (MM400, Retsch technology, Haan, Germany). Then, samples were centrifuged (600× *g* for 10 min at 4 °C) and for MDA spectrophotometric determination, aliquots of the supernatant were mixed with thiobarbituric acid (0.02 M) (1:1) and heated in boiling water for 20 min. After chilling the absorbance was measured spectrophotometrically at 532 nm (ScanGo, ThermoFisher Scientific, Alcobendas, Spain). Analyses were performed in duplicate. For quantification a standard curve was built with 1,1,3,3-Tetraethoxipropane (TEP). Results were expressed as mg MDA/kg muscle.

#### 2.2.4. Concentration of Vitamin E in Muscle

Concentration of α-tocopherol in muscle samples were determined by the procedure described by Rey et al. [14] in duplicate samples. Samples were homogenized in presence of a buffer (0.054 M dibasic sodium phosphate) adjusted to pH 7.0. Hexane was added to the tubes and after mixing and centrifugation (600× *g* during 10 min at 4 °C), the upper layer was collected and evaporated to dryness. Tocopherol was dissolved in ethanol prior to analyses by reverse phase HPLC (HP 1100, equipped with a diode array detector and a reverse phase RP-C18 column) (Agilent Technologies, Waldbronn, Germany) [14]. α-Tocopherol was quantified by means of a standard curve built using the pure compound (Sigma, Alcobendas, Madrid) and results were expressed as µg of α-tocopherol per g of muscle. 

#### 2.2.5. Extraction of Total Intramuscular Fat and Free-Fatty Acid Fraction

Meat samples (previously lyophilized) were weighed in a safe-lock micro test tube, and after adding dichloromethane-methanol (1.5 mL) (8:2), the mixture was mixed in a mixer mill (MM400, Retsch technology, Haan, Germany) [17] and centrifuged for 8 min at 10,000 rpm (Hermle Z383K, Thermo Fisher, Alcobendas, Madrid, Spain). Then, the upper layer was evaporated under nitrogen stream and lipid content was collected. Extraction of free fatty acids (FFA) fraction was made in 20 mg of intramuscular fat using the procedure described by Ruíz et al. [18] by means of aminopropyl minicolumns (Varian, Harbor City, CA, USA). FFA were obtained with the addition of diethylether:acetic acid (5 mL) (98:2) to the columns that contained fat previously dissolved in a mixture of hexane:chloroform:methanol (95:3:2). Then, FFA fraction was esterified by heating (80 °C for 1 h) in presence of methanol:toluene:H_2_SO_4_ (88:10:2) [19]. The fatty acids methyl esters were extracted with hexane, and directly injected in a gas chromatograph (HP 6890 Series GC System; Hewlett Packard, Avondale, PA) equipped with an automatic injector (170 °C), a flame ionization detector held at 250 °C and a column HP-Innowax Polyethylene Glycol (30 m × 0.316 mm × 0.25 µm). The oven temperature raised to 210 °C at a rate of 3.5 °C/min, then to 250 °C at a rate of 7 °C/min and was held constant for 1 min. Peaks were identified by comparing their retention times with those of authentic standards (Sigma–Aldrich, Alcobendas, Spain) and results were expressed as g per 100 g quantified fatty acids.

#### 2.2.6. Muscle Proteolysis

The muscle proteolysis was evaluated by the myofibrillar fragmentation index (MFI) in duplicate as described by Culler et al. [20]. Each sample (4 g) was homogenized in presence of cold MFI buffer (100 mM KCl, 20 mM potassium phosphate at pH 7, 1 mM MgCl_2_, and 1 mM NaN_3_ in distilled deionized water) and centrifuged at 1000× *g* for 15 min at 2 °C. The pellet was resuspended in cold MFI buffer (40 mL) and centrifuged again. Finally, the pellet was resuspended in 10 mL cold MFI buffer and vortexed until well mixed. Then, the sample was poured through a polyethylene strainer to remove the connective tissue and the tube was rinsed with an additional cold MFI buffer (10 mL). To determinate the protein content in each suspension, the extract (0.25 mL) was mixed with MFI buffer (0.75 mL) and Biuret reagent (4 mL) (Sigma Aldrich, Alcobendas, Madrid, Spain) and after mixing was placed in the dark for 30 min. Bovine serum albumin (BSA) (Sigma Aldrich) was used as standard at concentrations of 0, 2.5, 5, 7.5, and 10 mg/mL. MFI measurement (approximately 0.5 mg protein/mL solution) was measured spectrophotometrically at 540 nm (Thermo Scientific Multiskan GO; Thermo Fisher, Alcobendas, Madrid, Spain) and it was expressed as absorbance of a myofibrillar protein solution multiplied by 200.

#### 2.2.7. Texture Analysis of Muscle

The textural profile analysis (TPA) of *Longissimus dorsi* samples were measured by means of a TA.XT 2i/SMS Stable Micro System Texture Analyzer (Stable Microsystems Ltd., Surrey, England) equipped with an informatics program Texture Expert. Measurements (four per sample) were carried out at 22 °C in muscle cuts (1.5 cm in diameter and 1 cm thick). The following parameters were defined: hardness (Newtons: N) = maximum strength required to achieve compression; adhesiveness (Newtons × second: N × s) = area under the abscissa after the first compression; springiness (meters: m) = height the sample recovers between the first and second compression; cohesiveness = point to which the sample can deform before its rupture; gumminess (N) = force to disintegrate a sample of semi-solid meat by swallowing (hardness × cohesiveness); chewiness (Joules: J) = (hardness × springiness × cohesiveness).

#### 2.2.8. Structural Analysis by Magnetic Resonance Imaging (MRI)

MRI is a non-invasive and non-destructive technique that was used to obtain structural information on the myofibrillar components. The spin-lattice or longitudinal (T1) and spin-spin or transverse (T2) relaxation times are potentially sensitive to local variations of water mobility and therefore can be used to assess food microstructure. Individual relaxation times, T1 and T2, were measured using Magnetic Resonance Imaging (MRI) (NMR unit of the Complutense University of Madrid, Spain). MRI experiments were carried out on a 1-Tesla MRI system (Icon 1T, Bruker BioSpin Gmbh, Ettlingen, Germany) that consisted of a permanent magnet and was equipped with a gradient system capable of reaching a 450 mT/m gradient. For measurements of the spin-spin or transverse relaxation time (T2), 70 spin-echo images were obtained from six meat samples at different echo times (TE = 5.5–385 ms). The remaining parameters were as follows: Number of averaged experiments (NA) = 1; Repetition Time (TR) = 6000 ms; Matrix size (MTX) = 240 × 160, Field of view (FOV) = 9 × 6 cm^2^. The data thus obtained were adjusted to an exponential equation: S (TE) = S_0_ exp ^(−TE/T2)^; where S (TE) is the image signal at each echo time (TE) and S_0_ is the signal when TE equals zero. 

For the measurements of the spin-net or longitudinal relaxation time (T1) 17 spin-echo experiments were acquired using different values of the repetition time (TR = 75–10,000 ms). The rest of the acquisition parameters remained constant between the different experiments and were the same as those used in the experiments of calculating the transverse relaxation time. The data thus obtained were adjusted to an exponential according to the equation: S (TR) = S_0_ [1-exp ^(−TR/T1)^]; where S (TR) is the signal of the image at each recovery time (TR) and S_0_ is the signal when the TR is infinite. The T1 and T2 relaxation times calculation were performed using the ParaVision 6.0.1 software (Bruker Biospin GmbH, Ettligen, Germany).

### 2.3. Statistical Analysis

Data were analyzed following a completely randomized design using the general linear model (GLM) procedure of SAS (version 9; SAS Inst. Inc., Cary, NC, USA). The experimental unit for analysis of data was the pig. To study differences in blood parameters, pH, drip loss, MDA, tocopherol, MFI, and free-fatty acid composition, dietary treatment was considered the fix effect. For texture parameters and longitudinal and transverse relaxation times, the fixed effects were the dietary treatment and the time. Data are presented as the mean ± standard error of the mean (SEM). Tukey test was used to detect significant differences at *p* < 0.05. Differences between means were considered statistically significant at *p* < 0.05. Pearson correlation analyses were carried out among MDA concentration of muscle and proportion of free PUFA fatty acids and between MDA and tocopherol concentration (Statgraphecs-18 program). Linear adjustment between variables was carried using the Statgraphics-18 program.

## 3. Results

### 3.1. Experiment 1

The blood parameters and oxidative status of pigs at slaughter are presented in Table 1. The level of vitamin D_3_ in serum was not significantly different between the experimental groups; however, serum calcium concentration was greater in pigs receiving vitamin D_3_ in water compared to the control group. Moreover, the concentration of cortisol tended to be lower (*p* = 0.097) in pigs supplemented with vitamin D_3_ compared to the control group. Concerning the oxidative status, no differences were detected in the level of serum MDA. The α-tocopherol concentration of serum samples did not differ between the experimental groups. However, pigs provided the vitamin D_3_ in water had numerically higher values of α-tocopherol in serum. 

In meat, significant differences were observed for the α-tocopherol content; in pigs supplemented with vitamin D_3_ a 40% increase was observed (*p* = 0.0004) compared to the control group. The increase in α-tocopherol possibly resulted in a tendency for lower muscle MDA values in meat samples from vitamin D_3_ supplemented pigs on days 5 and 8 (*p* = 0.10). Muscle total MDA production was also inversely correlated with muscle α-tocopherol (*r* = −0.52), and a linear adjustment between these variables was statistically significant (*p* = 0.012; *R^2^* = 0.27). In addition, meat from pigs supplemented with vitamin D tended to have a lower drip loss percentage (*p* = 0.070) when compared to the control group, although pH at 40 h was not significantly affected. 

**Figure 1:**
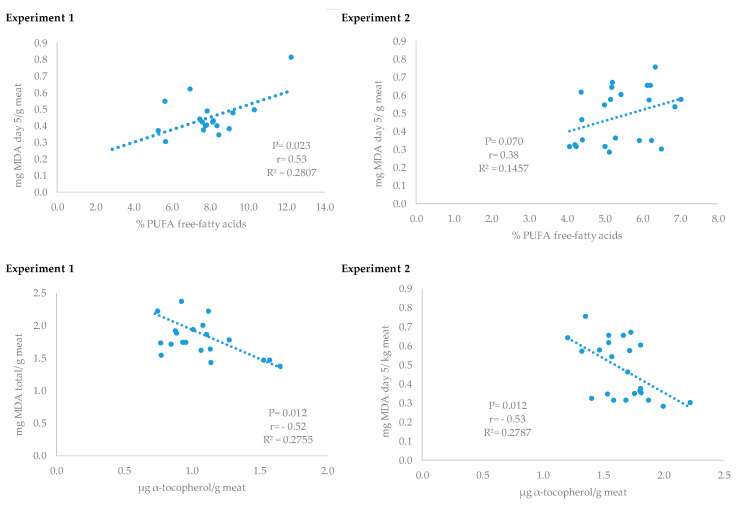
Relationship between thiobarbituric reactive substances (TBARS) values and muscle PUFA free-fatty acids proportion or α-tocopherol concentration from pigs supplemented with vitamin D_3_ or without supplementation prior to slaughter (experiments 1 and 2).

Since differences in TBARS production were not clear, the production of the specific fatty acid profile of free fatty acids was determined (Table 2). Pigs receiving the supplement had lower C18:2n-6 (*p* = 0.039), C16:1n-9 (*p* = 0.013), and C20:1n-9 (0.035); whereas the proportion of C16:0 (*p* = 0.047) and C17:0 (*p* = 0.007) was higher when compared to the control group. These differences in specific free fatty acids resulted in lower total free polyunsaturated fatty acids (*p* = 0.043), and a tendency to have higher total saturated fatty acids (*p* = 0.072) in the group receiving vitamin D_3_ in drinking water when compared with those without supplementation. The free unsaturated fatty acids that were mainly affected were n-6, whereas no statistical differences were found in n-3 or in monounsaturated fatty acids. In addition, the proportion of free PUFA fatty acids was directly correlated (*r* = 0.53) with MDA production at day 5 (Figure 1) and a linear adjustment (*p* = 0.023; *R^2^* = 0.28) was observed between both variables. 

The effect of vitamin D_3_ supplementation in drinking water on texture parameters of muscle was also evaluated (Table 3). Muscle from pigs receiving vitamin D_3_ in water had lower cohesiveness (*p* = 0.013), gumminess (*p* = 0.029), and chewiness (0.0001) and tended to have lower hardness (*p* = 0.08) and springiness (*p* = 0.054), when compared to the control group. However, adhesiveness was not affected. All these parameters were affected by time (*p* = 0.0001), although the interaction of treatment and time effects was not statistically significant.

### 3.2. Experiment 2

A second experiment was carried out to evaluate the effects of vitamin D_3_ dosage (Table 4). As observed in experiment 1, vitamin D_3_ (25-OH) levels in serum were also not affected by vitamin D_3_ supplementation, but cortisol decreased in vitamin D-supplemented pigs (*p* = 0.0130). At the same time, the α-tocopherol level and MDA production in serum were significantly affected (*p* = 0.011 and *p* = 0.014, respectively) by vitamin D supplementation; α-tocopherol was increased by 40% and MDA levels were decreased by 10%. No significant differences were observed in these parameters as an effect of the dose of vitamin D_3_ supplementation, and similar results were observed in both groups when compared to the control group.

Muscle parameters were also affected as an effect of vitamin D_3_ supplementation in this second experiment. Hence, muscle from pigs provided with vitamin D_3_ had lower water losses at 24 h (*p* = 0.030) and tended to have lower water losses at 48 (*p* = 0.06), although pH values were not significantly affected. Moreover, as observed in experiment 1, muscle α-tocopherol levels were higher after 8 days of refrigerated storage (*p* = 0.049) and tended to be higher at the beginning of the storage time (*p* = 0.12) in groups receiving vitamin D_3_ in drinking water. At the same time, MDA content was lower in meat from pigs receiving vitamin D_3_ after 5 days of refrigerated storage (*p* = 0.012). As indicated in experiment 1, a relationship was found between the concentration of tocopherol and MDA values of the muscle (*p* = 0.07; *r* = 0.38; *R^2^* = 0.14). Finally, the myofibrillar fragmentation index as a measurement of proteolytic activity was not statistically affected by vitamin D_3_ supplementation. No significant differences were observed concerning the dose of vitamin D_3_; apart from α-tocopherol that had intermediate values in pigs receiving 500,000 IU/L when compared to those supplemented with 700,000 IU/L.

The profile of free fatty acids is presented in Table 5. Pigs receiving the low-dose supplement had lower C18:2n-6 (*p* = 0.017), C16:1n-9 (*p* = 0.013), and total PUFA (0.008) when compared to the control group. As observed in experiment 1, the free unsaturated fatty acids that were mainly affected were n-6 (*p* = 0.005), whereas no statistical differences were found in n-3 or in monounsaturated fatty acids. In addition, the proportion of free PUFA fatty acids was directly correlated (*r* = 0.38) with MDA production at day 5 (Figure 1) and a linear adjustment (*p* = 0.07; *R^2^* = 0.14) was observed between the PUFA free-fatty acids and the muscle MDA concentration (Figure 1).

The effect of vitamin D_3_ supplementation in drinking water on texture parameters of muscle was also evaluated in experiment 2 (Table 6). As observed in experiment 1, muscle from pigs receiving vitamin D_3_ in water had lower cohesiveness (*p* = 0.006), gumminess (*p* = 0.043), and chewiness (0.007) and tended to have lower springiness (*p* = 0.089), when compared to the control group. Moreover, adhesiveness was also affected (*p* = 0.044). All these parameters, except cohesiveness were affected by time (*p* < 0.05), although the interaction of treatment and time effects were not statistically significant.

The imaging study of muscle by MRI revealed changes in relaxation times of different intensity with dietary treatment and sampling time (Table 7). Hence, transverse relaxation time (T2) was lower in muscle from pigs supplemented with vitamin D_3_ (*p* = 0.025); whereas longitudinal relaxation time (T1) was not statistically affected but tended to be greater in muscle from the control group (*p* = 0.10). Significant changes were also observed in T1 and T2 with meat conservation time. Hence, T1 decreased after 7 days of refrigerated display and T2 after 2 days (*p* = 0.0017 and *p* = 0.0005, respectively), resulting in higher intensity changes in T2 than in T1. The interaction between vitamin supplementation and conservation time was not significant and no differences were observed as an effect of the dose of vitamin D supplementation.

## 4. Discussion

The strategy of supplementing pigs’ drinking water with vitamin D_3_ prior to slaughter during fasting was evaluated in the present research in two experiments, since results may depend not only on the supplementation dose but also on the pigs’ water intake in relation to climatic conditions. The intake of amounts above 500,000 IU/day of vitamin D_3_ into drinking water decreased serum cortisol and increased α-tocopherol. Stressful situations such as fasting conditions prior to slaughter have been reported to increase cortisol levels in relation to the changes in certain nutrients with regulatory functions in the hypothalamic-pituitary-adrenal axis [21,22]. The effectiveness of vitamin D supplementation to reduce serum cortisol has been reported in some studies in humans [4,23] because both compounds compete for similar receptor sites [5]. However, to the best of our knowledge, there is a lack of information on the possible effect of its application in animal production practices. In suckling piglets after a single oral administration of 40,000 IU at 2 days of age, it was observed that these were heavier than untreated piglets at weaning and 7 days post-weaning; and authors suggested that more studies were needed to understand this effect of vitamin D under stress conditions [11]. The present research shows that doses above 500,000 IU/animal are needed in fasting pigs to observe positive effects related with stress control. In previous studies in human adults with 2000 IU/day [23] or 4000 IU/day [4] and, in infants with 400 IU/day combined with sunlight (7–14 h/week) [24] decreased cortisol values were observed. However, supplementation period was 12–16 weeks in adults and 8 weeks in infants (resulting in lower total doses than those used in the present study). Moreover, stressful situations have been associated with a disruption of the antioxidant balance and decreased serum α-tocopherol concentrations [25]. In the present research, 500,000 IU of vitamin D_3_ short-term supplementation improved the oxidative status of fasted pigs as indicated by α-tocopherol values, and 800,000 IU/animal are needed to reduce MDA production. Many studies in humans reported this effect on oxidative stress in relation to vitamin D status [26]; however, there is a lack of information in animals under stressful conditions. Recent studies [7] reported that vitamin D_3_ supplementation given at different small doses up to a total of 560,000 IU during the experimental period (6 m) enhanced glutathione peroxidase enzyme in adults. In addition, it has been found that Ca^2+^ may prevent lipid oxidation in vitro [27] through its ability to interact with superoxide anion radicals, which is in line with the inverse relationship found between serum Ca^2+^ and MDA values in the present study. In addition, the administration of calcium plus vitamin D supplements (50,000 IU twice during the study) produced in humans a significant increase in GSH and prevented a rise in MDA concentration [28]. However, other authors have not found such a relationship when providing high levels of vitamin D_3_ in beef cattle [29]. Furthermore, according to our results, the higher serum concentrations of α-tocopherol observed in pigs that consumed at least 800,000 IU of vitamin D_3_ in drinking water prior to slaughter may explain in part the lower MDA production in supplemented pigs during fasting. Some studies reported the effectiveness of vitamin E to control oxidative stress in piglets under stressful conditions [25]. The previous literature reported that vitamin D reduces vitamin E uptake [30] because these vitamins share common transport proteins. However, these effects have been reported in animals receiving food and not in fasting conditions. According to the results of the present study, the physiological stress and oxidative status control in fasting conditions by adding vitamin D_3_ to water might have beneficial effects on vitamin E serum preservation. Therefore, administration of vitamin D_3_ to drinking water prior to slaughter may be an interesting strategy to improve vitamin E levels.

The antioxidant effect of vitamin D_3_ inclusion into water was also observed in meat after 800,000 IU of vitamin D_3_/animal intake (experiment 2). Hence, meat from pigs supplemented with 500,000 IU/L or with 700,000 IU/L, with a consumption of at least 800,000 IU/animal had lower MDA levels on day 5 of refrigerated storage. These groups also had higher levels of α-tocopherol in meat and these values were inversely correlated with meat oxidation; whereas no correlations were observed between meat MDA and serum vitamin D levels. Other authors have reported the relationship between blood α-tocopherol levels and α-tocopherol accumulation in tissues [31,32] and the potent antioxidant effect of this vitamin on meat lipid oxidation [31,32,33]. However, there is a lack of information in the literature regarding the effects of vitamin D supplementation in drinking water on muscle α-tocopherol concentrations. Taking into account that stressful conditions, such as fasting, may increase cortisol levels [21,22], oxidative stress [22,34], and disrupt oxidant/antioxidant equilibrium [22,25], and the positive effect of vitamin D on controlling physiological stress [23] and antioxidant enzymes in vivo [7], the administration of this vitamin could also be of interest to preserve muscle tocopherol concentrations and meat stability. 

To the best of our knowledge, there is not much information on the possible effects of vitamin D_3_ supplementation on meat lipid oxidation. One of the studies carried out on beef [29] found a pro-oxidant effect of vitamin D_3_ supplementation in the diet when using doses of 7 million IU/day/animal for 3 or 6 days or 1 million IU/day/animal for 9 days. This is significantly higher than the lowest dose used in the present study per animal (500,000 IU) in short-term administration. However, another study carried out in pigs [35] reported increased antioxidant capacity in meat from pigs receiving 50 µg of vitamin D_3_/kg of feed (2 million IU/kg) for 55 days, and authors explained this antioxidant effect by the higher DPPH antioxidant activity of this form of vitamin. Lahucky et al. [36], reported a slight antioxidant effect in pigs receiving 500,000 IU of vitamin D_3_/day, compared with a control group or with a group supplemented with vitamin E, although the supplementation time for the vitamin D_3_-supplemented group was 5 days (lower than the vitamin E supplemented animals). The lack of significant effects on oxidation as an effect of the supplementation dose (500,000 IU/L vs. 700,000 IU/L) of the present study could be possibly explained either by the saturation effect when vitamin D_3_ was solubilized in water or more likely by the period of supplementation. A peak absorption in plasma at 6 h with an absorption range between 45% and 100% 3 h after the oral dose, and a net mean value of 78.6% with supplementation doses of 0.5–1 mg of Vitamin D_3_ has been reported in humans [37]. In pigs, an absorption of 50% of vitamin D administered orally has been documented [38]. However, the increase in vitamin D_3_ absorption depends on the basal levels [39,40]. Therefore, the higher the basal levels the lower the absorption. This leads us to believe that the use of high doses or prolonged administration may not produce the expected positive effects. Hence, Tipton et al. [41] suggested vitamin D_3_ supplement withdrawal some days before slaughter to achieve improvements in meat quality. Another important observation of the results presented is that the antioxidant effect of vitamin D_3_ supplementation was more significant in experiment 2 than in experiment 1. These experiments were carried out at different times and there was different water intake because of climate conditions (higher temperatures during the second experiment than in the first one). Moreover, the sex of the animals could affect, in part, the more significant results observed in the second experiment, since it has been reported that the favorable increase in glutathione peroxidase of vitamin D_3_ supplementation was mainly found in males [7]. The oxidative status of meat was also evaluated by the quantification of the specific free fatty acid formation. An excess of lipid oxidation induces the formation of more free fatty acids due to lipolysis and hydrolytic changes [34], and depending on the specific free fatty acid proportions meat sensory characteristics may also be affected [42]. In this sense, some antioxidants have shown anti-lipase activity and consequently are able to control lipid stability [43,44]. In the present study lower free PUFA production was observed in meat from pigs that consumed at least 500,000 IU of vitamin D when compared to the control group. In addition, both experiments showed a direct relationship between free PUFA fatty acid proportion and meat MDA concentration. A similar relation between free PUFA and TBARS production has been found in other studies [34]. It has been reported that vitamin D may act on lipid metabolism [45] and can rapidly accumulated in adipose tissue [46]. However, there is no information of their possible effects on lipolysis in tissues. The higher vitamin E concentration found in meat from vitamin D supplemented pigs could contribute to control lipid stability and free fatty acid production [44]. Moreover, an antilipolytic effect of Ca^2+^ has been reported in human adipocytes mainly through the inhibition of phosphodiesterase which would result in lipolysis inhibition [47]. The major contributors to rancidity and lipid oxidation development are polyunsaturated fatty acids [48], which would result in different concentrations of flavor precursors and development [42,49]. Consequently, vitamin D_3_ supplementation prior to slaughter could be an interesting strategy to improve the sensory characteristics of the products.

Concerning the proteolytic effect of vitamin D_3_ supplementation in water prior to slaughter, results on MFI or drip loss in the present study indicate that the dose of 500,000 IU of vitamin D_3_/l and intake of at least 800,000 IU/d under fasting would induce only minor effects on protein disruption. Drip loss was the meat quality characteristic most affected in relation to proteolytic activity, since both parameters were directly related, contrary to the inverse relation between drip loss and muscle pH [50,51]. Hence, meat from pigs that received vitamin D_3_ supplementation tended to have higher water holding capacity although no changes were detected in terms of pH. Other authors found improved drip loss and tenderness in chicken after 100,000 IU of supplementation for 7 days [52] and in steers [53] using doses from 7 to 9 million for 3 or 9 days, respectively. However, in pigs Wilborn et al. [12] only found a tendency for lower drip loss in animals supplemented with the highest dose (80,000 IU/kg feed) of vitamin D_3_ for 44 days; whereas a shorter period (10 days) but higher dose (175,000 IU/kg) resulted in a lack of effects of drip loss in pork [54]. Nevertheless, these authors found an increase in the pH range in barrows but not in gilts when compared to the control group. The use of lower doses (500,000 IU/day) for 5 days also resulted in non-significant differences in drip loss or pork pH [36]. The effects of vitamin D on proteolysis and indirectly on water preservation of meat has been related with increased serum Ca^2+^ levels [10] that activate the calpain proteases system. This explanation would be in line with the results presented in this study, in which pigs receiving vitamin D_3_ in water had higher Ca^2+^ concentrations. Moreover, other authors have found that muscle with greater α-tocopherol resulted in a higher proteolytic potential [51] which is also related to the greater muscle levels of the vitamin D-supplemented groups. Concerning the texture profile, in the present study, cohesiveness, gumminess, and chewiness decreased, in the VITD-500 group when compared to the control group (in both experiments). These texture characteristics have been reported to be decreased with ageing, a fact that reflected the progressive softness of the meat [55] and it has been inversely correlated with initial tenderness, and rate of breakdown [56]. Consequently, these results would indicate the positive effect on texture parameters of a short-term administration under fasting of at least 500,000 IU D_3_ per animal. To our knowledge, this is the first time that the effects of vitamin D_3_ supplementation in drinking water on tenderness in fasted pigs are examined. Other authors have not found effects on tenderness when using higher doses for a longer time in feed of pigs [12,35,36,54,57]. However, these authors applied different times of feeding withdrawal prior to slaughter and, in addition, tenderness was quantified by Warner–Bratzler shear force or sensory evaluation, which have been considered less robust measurements than the texture profile analysis [56]. Conversely, when supplementing vitamin D in feed in beef, different studies [10,58] reported changes in tenderness when assessed using the same methods used for pork, by Warner–Bratzler or sensory evaluation. In an interesting study carried out in cattle [41], the oral administration of 3 million IU/d for 5 days did not modify serum Ca^2+^ concentration immediately following supplementation or tenderness at day 0. However, after 7 days of supplement withdrawal serum Ca^2+^ increased and muscles from animals supplemented with vitamin D_3_ had lower Warner–Bratzler shear force values than the muscles from non-supplemented. Conversely, in pigs, Wiegand et al. [57] reported that the effective dose needed to raise blood plasma calcium concentration was at least 500,000 IU D_3_/d, and this dose applied for a short-term period (3 days) maintained plasma calcium for the longest time after cessation of vitamin D_3_ feeding. However, these authors did not find significant differences in tenderness even though the calcium increased. These effects suggest that regulation of calcium transport from plasma to muscle and its hormonal control deserves further investigation in pigs. 

Furthermore, a structural muscle magnetic resonance imaging analysis was carried out to study the possible effects of the dose of vitamin D_3_ on water holding capacity and its subsequent effects on proteolysis. This analysis revealed that transverse relaxation time (T2) was shorter in muscle from pigs supplemented with vitamin D_3_ in water and these values also decreased with storage time. T2 is associated with the extent of the relationship between water and peripheral structures and water compartmentalization and it has been reported that T2 values strongly correlate with bulk water [59] and inversely with water holding capacity [60]. Hence, high T2 means a lower degree of water binding to structures and consequently higher drip loss as observed in the control group of the present study. It is not clear whether these effects are due to vitamin D_3_ antioxidant protection on other antioxidant vitamins or proteolytic enzymes such as calpain; since it has been reported that vitamin E and selenium may protect calpain oxidation and then enhance muscle proteolysis and water retention [40]. Even though no differences were observed in the MRI parameters after the slaughter of the animal (first 2 days), the treatment applied would not affect the retention of the free water and the associated drip losses. However, after these first losses, the integrity of the structures would provide greater retention of aqueous content.

## 5. Conclusions

To conclude, the addition of vitamin D_3_ to fasting pigs’ drinking water prior to slaughter at 500,000 IU/L for 4 h (800,000 IU/animal) alleviates physiological stress and increases α-tocopherol of serum and muscle, improving the oxidative status and lipid stability. This dose of vitamin D_3_ and administration time is also enough to improve drip loss of muscle and tenderness. The water compartmentation study confirms the effects observed by physicochemical measurement of water holding capacity. Further research is needed to understand the mechanism of action on some of these effects.

## Figures and Tables

**Table 1:** Blood parameters and muscle quality measurements from pigs supplemented with vitamin D_3_ or without supplementation prior to slaughter (experiment 1).

Measurements	Control	VitD	SEM ^1^	*p* ^2^
***Blood parameters***				
Vit D_3_, 25-(OH) (ng/mL)	79.25	80.94	2.739	0.6768
Ca (mmoles/L)	3.39 ^a^	3.49 ^a^	0.024	0.0155
Cortisol (µg/dL)	7.49	6.29	0.484	0.0971
α-Tocopherol (µg/mL)	1.27	1.44	0.081	0.1952
TBARS (mmols MDA/L)	0.025	0.025	0.001	0.9379
***Longissimus dorsi muscle***				
pH, 40 h	5.45	5.42	0.036	0.4132
Drip loss at 72 h (%)	7.69	6.34	0.524	0.0706
α-Tocopherol (µg/g)	0.87 ^b^	1.23 ^a^	0.063	0.0004
MDA day 0 (mg/g)	0.31	0.37	0.034	0.2208
MDA day 3 (mg/g)	0.50	0.44	0.046	0.3442
MDA day 5 (mg/g)	0.51	0.42	0.039	0.1038
MDA day 8 (mg/g)	0.74	0.54	0.088	0.1053

^1^ SEM: mean standard error, *n* = 10; ^2^ *p*: differences were statistically significant when *p* < 0.05; values with different superscript (a, b) were statistically significant.

**Table 2:** Free-fatty acid proportion (g/100 g fatty acids) in muscle from pigs supplemented with vitamin D_3_ or without supplementation prior to slaughter (experiment 1).

% Free-Fatty Acid	Control	VitD	SEM ^1^	*p* ^2^
**C14:0**	1.330	1.230	0.066	0.2893
**C16:0**	24.134 ^b^	25.777 ^a^	0.559	0.0467
**C16:1n-7**	3.264	3.075	0.162	0.4099
**C16:1n-9**	0.521 ^a^	0.401 ^b^	0.032	0.0131
**C17:0**	0.279	0.379 ^a^	0.024	0.0076
**C17:1**	0.249	0.277	0.020	0.3325
**C18:0**	17.343	18.620	0.580	0.1274
**C18:1n-7**	3.957	3.896	0.271	0.8718
**C18:1n-9**	39.558	38.990	0.632	0.5228
**C18:2n-6**	8.238 ^a^	6.308 ^b^	0.630	0.0389
**C18:3n-3**	0.184	0.180	0.017	0.8671
**C18:4n-3**	0.092	0.095	0.006	0.6926
**C20:0**	0.121	0.117	0.011	0.7994
**C20:1n-9**	0.731 ^a^	0.655 ^b^	0.024	0.0354
**∑SAT** **^3^**	43.206	46.123	1.110	0.0724
**∑MUFA** **^4^**	48.280	47.294	0.742	0.3479
**∑PUFA** **^5^**	8.514 ^a^	6.583 ^b^	0.643	0.0426
**∑n-6** **^6^**	8.238 ^a^	6.308 ^b^	0.630	0.0389
**∑n-3** **^7^**	0.276	0.275	0.020	0.9808

^1^ SEM: mean standard error, *n* = 10; ^2^ *p*: differences were statistically significant when *p* < 0.05; values with different superscripts (a, b) were statistically significant; ^3^ SAT: sum of saturated fatty acids; ^4^ MUFA: sum of monounsaturated fatty acids; ^5^ PUFA: sum of polyunsaturated fatty acids; ^6^ ∑n-6: sum of n-6 polyunsaturated fatty acids; ^7^ ∑n-3: sum of n-3 polyunsaturated fatty acids.

**Table 3:** Texture parameters in muscle from pigs supplemented with vitamin D_3_ or without supplementation prior to slaughter (experiment 1).

Parameters	Control	VitD	Day0	Day5	Day8	SEM ^1^	SEM Time ^2^	P Treatment ^3^	P Time ^4^	P Treatment × P Time
**Hardness, N** **^5^**	44.10	40.10	47.60 ^a^	48.55 ^a^	29.49 ^b^	1.490	1.857	0.0799	0.0001	0.2689
**Adhesiveness, Nxs** **^6^**	−0.48	−0.51	−0.36 ^a^	−0.57 ^b^	−0.57 ^b^	0.020	0.025	0.3487	0.0001	0.4898
**Springiness, m** **^7^**	0.003	0.003	0.005 ^a^	0.001 ^c^	0.002 ^b^	0.000	0.000	0.0539	0.0001	0.2734
**Cohesiveness**	0.42 ^a^	0.40	0.36 ^b^	0.43 ^a^	0.44 ^a^	0.005	0.007	0.0134	0.0001	0.1670
**Gumminess, N**	18.13 ^a^	15.97 ^b^	17.08 ^b^	20.79 ^a^	13.06 ^c^	0.674	0.852	0.0294	0.0001	0.1544
**Chewiness, J** **^8^**	0.05 ^a^	0.04 ^b^	0.08 ^a^	0.03 ^b^	0.03 ^b^	0.003	0.004	0.0005	0.0001	0.4936

^1^ SEM: mean standard error the treatment effect, *n* = 30; ^2^ SEM time: mean standard error of the time effect, *n* = 20; ^3^ P treatment: *p* value for treatment effect; ^4^ P time: *p* value for time effect; ^3,4^ differences were statistically significant when *p* < 0.05; values with different superscript (a, b) were statistically significant; ^5^ N: Newtons; ^6^ Nxs: Newtons x second; ^7^ m: meters; ^8^ J: Joules.

**Table 4:** Blood parameters and muscle quality measurements from pigs supplemented with vitamin D_3_ at different doses (500,000 UI/L vs. 700,000 UI/L) or without supplementation prior to slaughter (experiment 2).

Measurements	Control		VitD-500		VitD-700		SEM ^1^	*p* ^2^
***Blood parameters***								
VitD_3_, 25-(OH) (ng/mL)	59.34		61.40		57.43		3.374	0.7422
Ca (mmoles/L)	3.07	^b^	3.23	^a^	3.30	^a^	0.028	0.0001
Cortisol (µg/dL)	12.14	^a^	7.88	^b^	9.40	^b^	0.890	0.0130
α-Tocopherol (µg/mL)	1.80	^b^	2.51	^a^	2.35	^ba^	0.138	0.0110
TBARS (mmols MDA/L)	0.009	^a^	0.005	^b^	0.006	^b^	0.001	0.0143
***Muscle pH and drip loss***								
pH 40 h	5.68		5.71		5.79		0.018	0.3338
Water loss at 24 h (%)	3.92	^a^	2.82	^b^	2.44	^b^	0.003	0.0302
Water loss at 48 h (%)	5.77		4.60		4.11		0.004	0.0603
Drip loss at 72 h (%)	6.89		5.95		8.20		0.847	0.1929
***Muscle α-Tocopherol***								
α-Tocopherol day 0 (µg/g)	1.43		1.64		1.66		0.084	0.1244
α-Tocopherol day 8 (µg/g)	1.59	^b^	1.82	^ba^	1.87	^a^	0.079	0.0493
***Muscle TBARS (mg MDA/g)***								
MDA day 0 (mg/g)	0.21		0.21		0.21		0.016	0.6089
MDA day 3 (mg/g)	0.27		0.25		0.25		0.011	0.9928
MDA day 5 (mg/g)	0.65	^a^	0.48	^b^	0.38	^b^	0.060	0.0121
MDA day 8 (mg/g)	1.38	^a^	1.02	^ba^	0.85	^b^	0.154	0.0669
***MFI (myofibrillar fragmentation index)***								
MFI day 1	17.35		17.81		16.18		2.247	0.8701
MFI day 8	22.63		26.37		19.07		2.639	0.1726

^1^ SEM: mean standard error, *n* = 8; ^2^ *p*: differences were statistically significant when *p* < 0.05; letters with different superscript were statistically significant.

**Table 5:** Free-fatty acid proportion (g/100 g fatty acids) in muscle from pigs supplemented with vitamin D_3_ at different doses (500,000 UI/l vs. 700,000 UI/l) or without supplementation prior to slaughter (experiment 2).

% Free-Fatty Acid	Control	VitD-500	VitD-700	SEM ^1^	*p* ^2^
**C14:0**	1.492	1.487	1.462	0.029	0.7429
**C16:0**	25.741	26.246	26.474	0.291	0.2141
**C16:1n9**	0.386 ^a^	0.314 ^b^	0.321 ^b^	0.017	0.0128
**C16:1n7**	3.380	3.518	3.269	0.117	0.3399
**C17:0**	0.346	0.273	0.300	0.028	0.2019
**C17:1**	0.274	0.184	0.225	0.030	0.1347
**C18:0**	16.831	16.979	17.555	0.555	0.6287
**C18:1n9**	40.631	41.999	41.208	0.758	0.4533
**C18:1n7**	2.959	2.714	2.894	0.119	0.3382
**C18:2n6**	6.325 ^a^	4.702 ^b^	4.779 ^b^	0.409	0.0167
**C18:3n3**	0.372	0.333	0.309	0.035	0.4413
**C18:4n3**	0.143	0.120	0.119	0.015	0.4370
**C20:0**	0.248	0.262	0.241	0.017	0.6751
**C20:1n9**	0.873	0.882	0.843	0.040	0.7778
**∑SAT ^3^**	44.892	45.247	46.033	0.678	0.4884
**∑MUFA ^4^**	48.874	49.611	48.761	0.725	0.6722
**∑PUFA ^5^**	6.234 ^a^	5.155 ^b^	5.206 ^b^	0.246	0.0081
**∑n-6 ^6^**	5.742 ^a^	4.702 ^b^	4.779 ^b^	0.223	0.0055
**∑n-3 ^7^**	0.491	0.453	0.427	0.045	0.6071

^1^ SEM: mean standard error, *n* = 8; ^2^ *p*: differences were statistically significant when *p* < 0.05; values with different superscript (a, b) were statistically significant; ^3^ SAT: sum of saturated fatty acids; ^4^ MUFA: sum of monounsaturated fatty acids; ^5^ PUFA: sum of polyunsaturated fatty acids; ^6^ ∑n-6: sum of n-6 polyunsaturated fatty acids; ^7^ ∑n-3: sum of n-3 polyunsaturated fatty acids.

**Table 6:** Texture parameters in muscle from pigs supplemented with vitamin D_3_ at different doses (500,000 UI/l vs. 700,000 UI/l) or without supplementation prior to slaughter (experiment 2).

Parameters	Control	Vit-500	Vit-700	Day0	Day8	SEM ^1^	SEM Time ^2^	P Treatment ^3^	P Time ^4^	P Treatment x P Time
**Hardness, N ^5^**	25.55	24.22	24.48	31.41 ^a^	18.10 ^b^	0.670	0.540	0.3053	0.0001	0.9988
**Adhesiveness, Nxs ^6^**	−0.42 ^a^	−0.38 ^b^	−0.40 ^ba^	−0.35 ^a^	−0.45 ^a^	0.012	0.010	0.0444	0.0001	0.5826
**Springiness, m ^7^**	0.0004 ^a^	0.0003 ^b^	0.0003 ^ba^	0.0002 ^b^	0.0005 ^a^	0.000	0.000	0.0887	0.0001	0.2679
**Cohesiveness**	0.52 ^a^	0.49 ^b^	0.50 ^ba^	0.50	0.50	0.007	0.006	0.0059	0.8869	0.9454
**Gumminess, N**	13.23 ^a^	11.84 ^b^	12.21 ^ba^	15.76 ^a^	9.11 ^a^	0.413	0.345	0.0431	0.0001	0.9784
**Chewiness, J ^8^**	0.004 ^a^	0.003 ^b^	0.004 ^b^	0.003 ^a^	0.004 ^a^	0.000	0.000	0.0070	0.0011	0.4407

^1^ SEM: mean standard error the treatment effect, *n* = 20; ^2^ SEM time: mean standard error of the time effect, *n* = 30; ^3^ P treatment: *p* value for treatment effect; ^4^ P time: *p* value for time effect; ^3,4^ differences were statistically significant when *p* < 0.05; values with different superscript (a, b) were statistically significant; ^5^ N: Newtons; ^6^ Nxs: Newtons × second; ^7^ m: meters; ^8^ J: Joules.

**Table 7:** Mean values of the spin-net or longitudinal relaxation time (T1) and the spin-spin or transverse relaxation time (T2) from pigs supplemented with vitamin D_3_ in water at different doses (500,000 IU/L vs. 700,000 IU/L) or without supplementation prior to slaughter (experiment 2).

Relaxation time	T1. ms	T2. ms
**Control**	947.7	49.1 ^a^
**VitD-500**	923.9	47.6 ^b^
**VitD-700**	921.9	47.0 ^b^
**Day 2**	957.9 ^a^	50.7 ^a^
**Day 4**	949.7 ^a^	47.9 ^b^
**Day 7**	927.7 ^a^	47.0 ^b^
**Day 13**	889.4 ^b^	46.1 ^b^
**SEM ^1^**	9.91	0.56
**SEM time ^2^**	9.92	0.52
**P treatment ^3^**	0.1006	0.0247
**P time ^3^**	0.0017	0.0005
**P treatment × P time**	0.6759	0.9639

^1^ SEM: mean standard error of the treatment effect, *n* = 6; ^2^SEM time: mean standard error of the time effect, *n* = 8; ^3^ *p* treatment: differences were statistically significant when *p* < 0.05; values with different superscript (a, b) were statistically significant.

## Supplementary Materials

The following are available online at https://www.mdpi.com/2076-3921/9/6/559/s1, Table S1: Calculated major nutrients of the experimental diets.
