# Supplementary material for: Vitamin D3 Supplementation in Drinking Water Prior to Slaughter Improves Oxidative Status, Physiological Stress, and Quality of Pork
Source: Antioxidants (Basel). 2020 Jun 26;9(6):559. doi: 10.3390/antiox9060559 (PMC7346143; doi:10.3390/antiox9060559)
Supplement: Supplementary file 1 [file antioxidants-09-00559-s001.pdf]

antioxidants

*Article*

## Supplementary material

**Table S1.**- Calculated major nutrients of the experimental diets

| % | Experiment 1 | Experiment 2 |
|---|---|---|
| Dry matter | 89.19 | 89.88 |
| Gross Protein | 13.57 | 13.14 |
| Gross Fat | 4.78 | 5.05 |
| Linoleic acid | 1.24 | 1.11 |
| Gross fiber | 4.39 | 3.41 |
| FAD | 5.74 | 4.79 |
| FND | 14.11 | 12.93 |
| Starch | 49.14 | 46.98 |
| Starch+suger | 52.82 | 52.31 |
| Ash | 3.82 | 3.99 |
| Calcium | 0.42 | 0.32 |
| Phosphoro total | 0.37 | 0.40 |
| Lys | 0.77 | 0.75 |
| EN (mcal/kg) | 2.45 | 2.48 |
| ED (Mcal/kg) | 3.36 | 3.44 |

**Experiment 1**: Ingredients (%): Wheat, 35.00; Corn, 30.00; Bisquit, 8.53; Sunflower 35, 8.00; Rye, 7.46; Soya 47, 3.23; Rapeseed, 2.00; Recycled, 1.50; Fat 3/5 disc, 1.00; Fat 3/5 1.00; Calcium carbonate, 0.80; Lysine 50 liquide, 0.60; Salt ,0.40; Premix, 0.30; L-Threonine, 0.08; Surfactant acid, 0.05; Enzymes β-xilanase, 0.04; Phytase 5000 liquide 0.01. Premix in feed: Vitamin A 4 M UI/Kg; Vitamin $D_3$ 0.4 M UI/Kg 25-OH $D_3$; Vitamin E 40 mg/Kg; Vitamin $B_1$ 2 mg / Kg; Vitamin $B_2$ 2 mg/Kg; Vitamin $B_6$ 3 mg/Kg; Vitamin $B_{12}$ 0.02 mg/Kg; Vitamin $K_3$ 2 mg/Kg; Nicotinic acid 20 mg/Kg; Pantothenic acid 10 mg/Kg; Choline chloride 100 mg/Kg; Manganese oxide 40 mg/Kg; Zinc oxide 100 mg/Kg; Ferrous sulphate 300 mg /Kg; Copper sulphate 15 mg/Kg; Yodure 0.3 mg/Kg; Sodium selenite 0.40 mg/Kg.

**Experiment 2**: Ingredients (%): Triticale, 30.00; Wheat, 30.00; Bisquit, 10.00; Barley, 8.79; Soya 47, 8.53; Rice, 5.87; Recycled, 2.00; Fat 3/5 disc, 1.11; Fat 3/5, 1.00; Vegetable glycerol, 1.00; Calcium carbonate, 0.56; Lysine 50 liquide, 0.43; Salt, 0.40; Premix, 0.20; L-Threonine, 0.08; Surfactant acid, 0.05; DL-methionine, 0.03; Enzymes β-xilanase, 0.02; Phytase 5000 liquide, 0.01.; Premix in feed: Vitamin A 5 M UI/Kg; Vitamin $D_3$ 1.0 M UI/Kg 25-OH $D_3$; Vitamin E 30 mg/Kg; Vitamin $B_1$ 2.0 mg/Kg; Vitamin $B_2$ 2.0 mg/Kg; Vitamin $B_6$ 3.0 mg /Kg; Vitamin $B_{12}$ 0.020 mg/Kg; Vitamin $K_3$ 2.0 mg/Kg; Nicotinic acid 20 mg/Kg; Pantothenic acid 10 mg/Kg; Choline chloride 100 mg/Kg; Manganese oxide 40 mg/Kg; Zinc oxide 100 mg/Kg; Ferrous sulphate 90 mg/Kg; Copper sulphate 15 mg /Kg; Yodure 0.5 mg/Kg; Sodium selenite 0.40 mg/Kg

© 2019 by the authors. Submitted for possible open access publication under the terms and conditions of the Creative Commons Attribution (CC BY) license (http://creativecommons.org/licenses/by/4.0/).
